# Grating-Corner-Cube-Based Roll Angle Sensor

**DOI:** 10.3390/s20195524

**Published:** 2020-09-27

**Authors:** Siyu Zhou, Vunam Le, Qinggai Mi, Guanhao Wu

**Affiliations:** State Key Laboratory of Precision Measurement Technology and Instruments, Department of Precision Instrument, Tsinghua University, Beijing 100084, China; zhousy17@mails.tsinghua.edu.cn (S.Z.); lwn17@mails.tsinghua.edu.cn (V.L.); miqinggai@mail.tsinghua.edu.cn (Q.M.)

**Keywords:** roll angle, grating-corner-cube sensor, diffraction and reflection characteristics, differential detection

## Abstract

This paper presents a specifically designed grating-corner-cube sensor for precise roll angle measurements. Owing to the diffraction characteristics of the transmission grating and reflection characteristics of the corner cube, two spatially separated parallel beams are naturally constructed. Through differential detection of the positions of two parallel beams, we experimentally demonstrate the possibility of a precise roll angle measurement at a high refresh rate. A performance evaluation of the proposed technique indicates a stability of 0.46 arcsec over 5 min. Compared with a commercial autocollimator over a range of 500 arcsec, the residuals are maintained within ±2 arcsec with a standard deviation of 1.37 arcsec. Furthermore, a resolution of 0.8 arcsec can be achieved using the proposed method. The developed compact roll angle sensor has potential applications in academic and industrial fields.

## 1. Introduction

In recent years, there has been an increasing demand for attitude determination of the target in remote sensing [1], spacecraft rendezvous and docking [2], and high-end equipment manufacturing, including lithography machines [3], coordinate measuring machines [4], and numerically controlled machine tools [5]. These applications require precise and dynamic attitude measurements and feedback adjustment [6]. Attitude measurements include measurements of the pitch, yaw, and roll angle [7]. Among them, the roll angle measurement is crucial and challenging.

Several non-contact roll angle measurement methods have been reported, including the polarization–modulation method [8,9,10], interference method [11,12,13,14,15], and differential position measurement method [16,17,18,19,20,21]. The principle behind the polarization–modulation method involves the modulation of the polarization state of the measurement beam through roll angle variation, wherein the roll angle can be calculated based on intensity measurements. Gillmer et al. reported an accuracy of 28.4 μrad over a 50-mrad range and a resolution of 40 μrad over a range of 0.75 rad [9]. Although this polarization–modulation method offers a large measurement range, it does not achieve sufficient resolution. Compared with other methods, the interference method is highly sensitive owing to its precise interferometric phase measurements. Qi et al. proposed a resolution-enhanced interferometer for roll angle measurements [14]. Using the reported method, a resolution of 0.13 arcsec was achieved with an accuracy within ±4 arcsec in a range of ±360 arcsec. However, the interference method can only achieve incremental measurements and requires a relatively complex optical configuration. The differential position measurement method is the most popular method employed in industrial fields, wherein the roll angle can be easily measured based on position detection with a relatively high accuracy and high response speed. The generation of two beams for differential detection is a critical aspect of this method. In general, as two corner cubes are used as a combined target, the installation of these cubes is necessary, with the requirement for a large base to fix the targets, which encumbers the system [17]. In addition, the measurement accuracy of the roll angle is easily affected by the crosstalk error of the other degrees of freedom and the installation error of the two corner cubes [18]. To improve the compact performance of the system, novel targets have been designed to generate two spatially separated beams [19,20]. For example, using a compact rhombic prism as the target, two parallel beams can be naturally generated. This method provided a resolution of 0.25 arcsec, and the maximum error was 3.5 arcsec over a range of 160 arcsec [19]. Notably, the effects of fabrication and installation errors, which lead to non-parallelism of the two beams, should be considered. In an early study by Gao et al., a reflection grating, instead of a plane mirror, was used as the target in the autocollimation method [21]. Using a simple target, the roll angle can be calculated by simultaneously detecting the positions of the zero- and first-order diffracted beams. However, the diffracted beams are not parallel to the incident beam, and thus, they have limited applications in the case of long stand-off distances.

In this letter, we propose a grating-corner-cube (GCC)-based roll angle sensor. It combines the diffraction characteristics of the transmission grating and reflection characteristics of the corner cube. After passing through the GCC sensor, the incident beam is diffracted twice by the grating and reflected once by the corner cube. The two spatially separated beams are naturally constructed and exit the sensor parallel to the incident beam. As the directions of the positive and negative first-order diffracted beams are opposite to each other and both change with the direction of the grating period, the roll angle of the target can be accurately determined by differentially detecting the positions of the two parallel beams.

The rest of the paper is organized as follows. In Section 2, the experimental setup and principle of the proposed roll angle measurement method are described. A theoretical model of the GCC sensor is also presented. In Section 3, comparison, stability, and resolution experiments are presented to evaluate the feasibility of the proposed method. In Section 4, further investigations on the GCC sensor performance are presented. Lastly, the conclusions are presented in Section 5.

## 2. Experimental Setup and Principle

### 2.1. Experimental Setup

**Figure 1:**
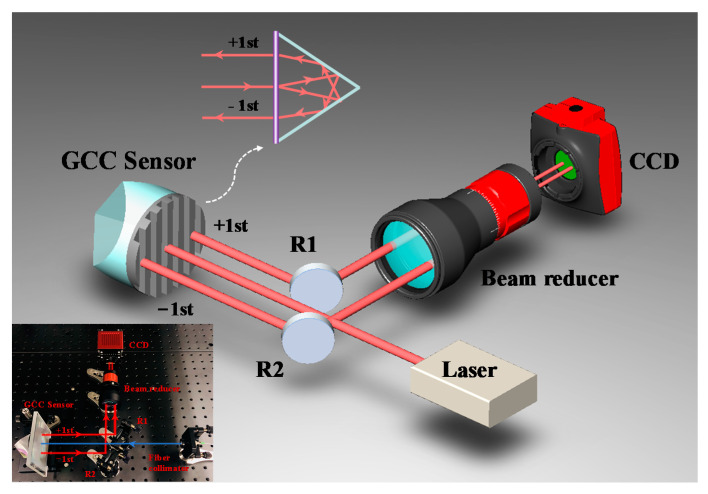
Schematic of the experimental setup. The magnification of the beam reducer is ≈2.7, which is used to control the beam diameter. The actual experimental setup is also depicted.

Figure 1 shows a schematic of the proposed roll angle measurement method. A collimated beam with a diameter of ≈3 mm emitted from a free-running mode-locked laser (center wavelength *λ*_c_ ≈ 1576.5 nm, maximum output power ≈ 50 mW) is normally incident onto the GCC sensor, which is composed of a corner cube and transmission grating. The incident beam is diffracted by the grating. The positive and negative first-order diffracted beams are reflected by the corner cube and finally exit the sensor parallel to the incident beam after diffraction by the grating again. The distance between the two beams depends on the diffraction angle and sensor size. After passing through two spatial parallel reflectors, R_1_ and R_2_, the two measurement beams are reflected to a beam reducer system and finally sampled by a near-infrared charge-coupled device (CCD; Goldeye, Allied Vision Company, pixel size: 15 μm).

When the roll angle of the target changes, the directions of the positive and negative first-order diffracted beams change with the periodic direction of the grating. The angle variation can be easily calculated using a mathematical model of the roll attitude of the target and spatial distribution of the two measurement beams. Considering the limitation imposed by the critical angle of the corner cube, the grating period (g) was set to 5 μm. The critical angle is the maximum incident angle that satisfies the retro-reflective properties of the corner cube. In this study, a transmission grating with an effective aperture of 50 mm was specifically designed and fabricated by a dual-beam exposure system [22]. The spacing error of the entire grating surface is better than 0.03 g, and the diffraction efficiency of the one-dimensional grating is ≈ 26%. The transmission grating is closely connected to the front surface of the corner cube (PS976-C, Thorlabs, Inc.; transmission of ≈90%) to enable the installation of a GCC sensor. Anti-reflection coatings with wavelengths ranging from 1050 to 1700 nm were applied to the input surface of the corner cube to avoid surface reflection. Two parallel measurement beams separated in space are naturally constructed via the GCC sensor.

### 2.2. Theoretical Model

**Figure 2:**
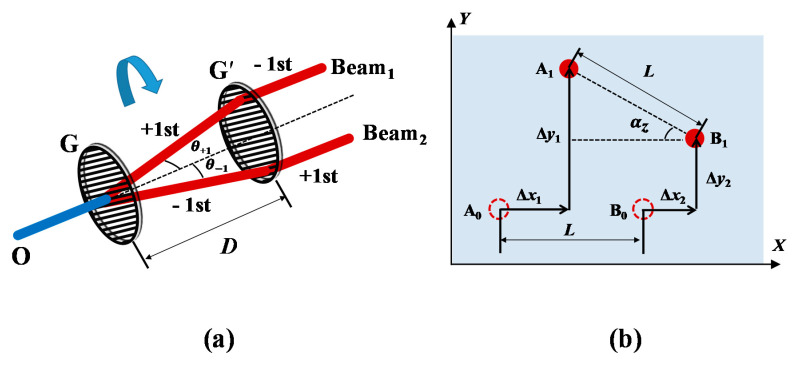
Principle of the proposed method. (**a**) Theoretical model; (**b**) Space distribution of the two measurement beams after angular motions and lateral movements.

As depicted in Figure 2a, the model of the GCC sensor can be regarded as a parallel grating pair (G and G′). Being twice diffracted by the sensor is equivalent to being twice diffracted by the grating G and its virtual image G′. The incident beam emitted from point O is diffracted by grating G and generates +1st- and −1st-order diffracted beams. Then, these two diffracted beams are diffracted by the grating G′. If the two diffracted orders of the beam passing through G and G′ are opposite to each other, the two parallel beams, beam 1 and beam 2, exit parallel to the incident beam. The spatial positions of beam 1 and beam 2, before and after the rotations and lateral motions, change from A_0_, B_0_ to A_1_, B_1_ (Figure 2b). The movements of beam 1 and beam 2 caused by the lateral motions of the target are equal. When the target exhibits a small roll angle *α*_z_, the directions of the ±1st-order diffracted beams change with the periodic direction of the grating. In this case, Δ*y*_1_ is not equal to Δ*y*_2_, and thus the roll angle can be expressed as

(1) $$\alpha_{z}=\frac{\Delta y_{1}-\Delta y_{2}}{L},$$

where *L* is the distance between beam 1 and beam 2, which is equal to *D*tan(*θ*_+1_)+ *D*tan(*θ*_−1_). *D* is the grating pair spacing, which is equal to twice the distance from the vertex of the corner cube to the front surface of the grating. The diffracted angle (*θ*_+1_ and *θ*_−1_) can be calculated according to the diffraction equation.

According to Equation (1), when the distance *L* is fixed, the precision of the roll angle depends mainly on that of the positions of the two beams. In the experiments, the mode-locked laser was used as the light source. To achieve high-precision measurements, a low-noise near-infrared CCD camera was employed as the detector. The displacements of beam 1 and beam 2 can be calculated by the centroid location of the two spots on the CCD. The centroid coordinate (*x*_c_, *y*_c_) can be calculated using the intensity-weighted average method.

(2) $$x_{c}=\frac{\sum_{y=1}^{m}\sum_{x=1}^{n}I(x,y)\times x}{\sum_{y=1}^{m}\sum_{x=1}^{n}I(x,y)},$$

(3) $$y_{c}=\frac{\sum_{y=1}^{m}\sum_{x=1}^{n}I(x,y)\times y}{\sum_{y=1}^{m}\sum_{x=1}^{n}I(x,y)},$$

where *I*(*x*,*y*) is the gray value of the corresponding pixel (*x*, *y*), and *m* and *n* are the pixel numbers of the light spot along the two dimensions. Due to the limitation imposed by the size of the sensitive area of the CCD, the spot size should be controlled. A beam reducer with a magnification of ≈2.7 was added to reduce the sizes of beam 1 and beam 2. As the two spots are sampled by the same detector, the centroid coordinates of the two spots can be obtained using the region segmentation method.

## 3. Experiments

An experimental setup was constructed to test the measurement performance. Its measurement accuracy, stability, and resolution were evaluated.

### 3.1. Comparison Experiments

**Figure 3:**
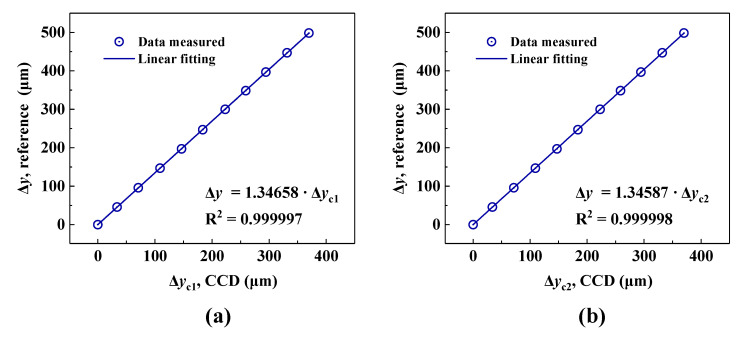
Linear calibration results. (**a**) Centroid displacement Δ*y*_c1_ versus Δ*y* obtained by the inductive sensor. (**b**) Centroid displacement Δ*y*_c2_ versus Δ*y* obtained by the inductive sensor.

According to Equation (1), Δ*y*_1_ and Δ*y*_2_ represent the linear displacements of beam 1 and beam 2, respectively. A magnification exists between the linear displacements of the target and the displacements of the spot sampled by the CCD caused by the beam reducer. The parallel error of the two reflectors, R_1_ and R_2_, also leads to measurement errors in the roll angle. To calibrate the magnification and compensate for the non-parallelism error of the dual beam, we compared the *y*-directional displacements of the two spots (Δ*y*_c1_ and Δ*y*_c2_) on the CCD and the *y*-directional displacement of the GCC sensor (Δ*y*) measured by an inductive sensor (Millimar 1240, Mahr, Inc, resolution: 10 nm). The *Y*-directional displacement was applied by a linear moving stage. The distance was changed to a total value of 500 μm using 10 steps at a stand-off distance of ≈1 m. For each position, the data were recorded for 1 s by the CCD, and the average centroid results Δ*y*_c1_ and Δ*y*_c2_ were used for the calibration, respectively. As depicted in Figure 3a,b, through linear fitting, the slopes of the two spots were determined to be 1.34658 and 1.34587, while the correlation coefficients (*R*^2^) were calculated to be 0.999997 and 0.999998, respectively. Notably, considering the reflection characteristics of the corner cube, the displacements of both beam 1 and beam 2 are twice that of the GCC sensor. Therefore, the displacements of beam 1 and beam 2 (Δ*y*_1_ and Δ*y*_2_), according to Equation (1), can be expressed as Δ*y*_1_ = 2 × 1.34658 × Δ*y*_c1_ and Δ*y_2_* = 2 × 1.34587 × Δ*y*_c2_, respectively. After calibrating the magnification and compensating for the non-parallelism error of the dual beam, the roll angle can be easily obtained by centroid determination of the two spots.

**Figure 4:**
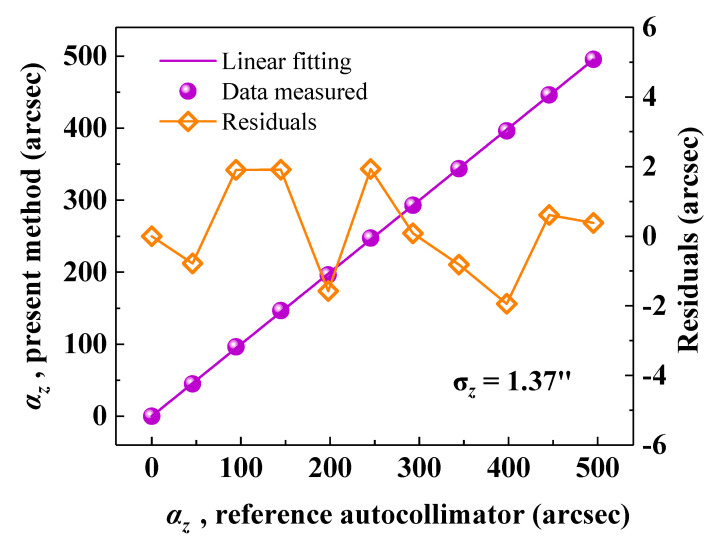
Angle results, *α*_z_, obtained by the proposed method versus the results obtained by the commercial autocollimator.

Moreover, to verify the measurement accuracy, the GCC sensor was mounted on a precision rotary stage. A commercial autocollimator (Collapex 200, AcroBeam, accuracy: 0.3 arcsec within ±600 arcsec) was used as the reference. The comparison range was 500 arcsec with a step of 50 arcsec. The roll angle was simultaneously measured using the proposed method and autocollimator. For each position, the data were recorded for 1 s, and the average results were used for comparison. As depicted in Figure 4, the results measured by the proposed method are basically consistent to those obtained by the commercial autocollimator. Using linear fitting, the slope and correlation coefficient (*R*^2^) were determined to be 1.0002 and 0.99993, respectively. The comparison residuals in the range of 500 arcsec are remained within ±2 arcsec, with a standard deviation (STD, *σ*_z_) of 1.37 arcsec. The comparison residuals can be mainly attributed to the random noise of the infrared CCD and air turbulence.

### 3.2. Stability Experiments

**Figure 5:**
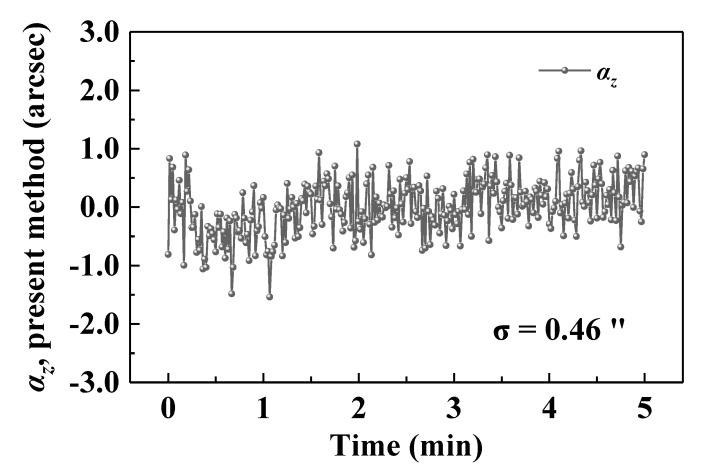
Experimental results of the roll angle stability. The stability was evaluated over 5-min data with 300 results.

Stability results were also obtained at a stand-off distance of ≈1 m. The environmental temperature was approximately 25.4 ± 0.1 °C. The experimental setup was fixed on an optical platform. The angle results were automatically extracted once every 1 s for 5 min to evaluate the stability of the proposed method. As shown in Figure 5, the jitter of *α*_z_ is distributed in the range of −1.53 to 1.08 arcsec with an STD (*σ*) of 0.46 arcsec. The random noise can be mainly attributed to the detector noise and stability of the laser beam, while the low-frequency drift is caused mainly by the environmental perturbations. Notably, although the environmental perturbations lead to a change in the beam direction, these perturbations associated with the two beams can be eliminated through differential measurements owing to the approximately common optical path of beam 1 and beam 2 in space. The response speed can reach 300 Hz when using the proposed method. Therefore, under relatively low-speed measurement conditions, random noise can be suppressed by averaging the results using the moving average method.

### 3.3. Resolution Experiments

**Figure 6:**
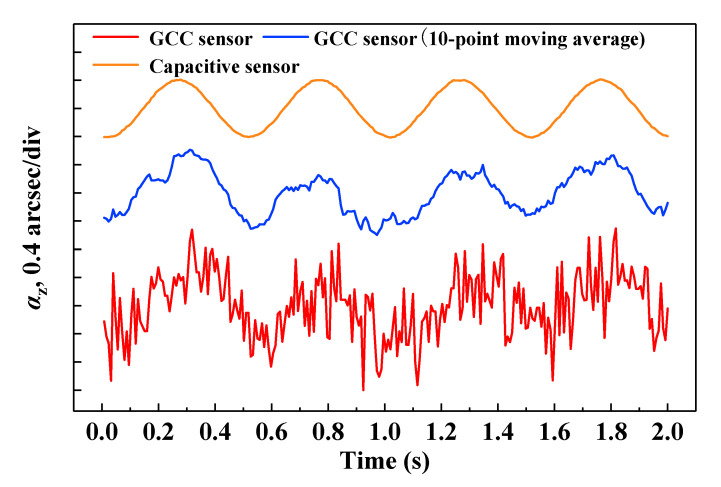
Resolution results of the roll angle at a modulation frequency of 2 Hz. The results obtained by the proposed method and capacitive sensor are presented.

To further verify the resolution of the proposed method, dynamic roll motion measurements were carried out using a piezo stage (P-562, PI, Inc.) in a closed-loop mode. At a stand-off distance of ≈1 m, the GCC sensor was mounted on the piezo stage and subjected to continuous roll motions at a modulation frequency of 2 Hz with an amplitude of 0.8 arcsec. The data were recorded for 2 s, and measurement results were obtained using the proposed method and capacitive sensors. As depicted in Figure 6, 0.8-arcsec continuous sinusoidal motions were observed through the proposed method, and the results were consistent with those measured by the capacitive sensors. As mentioned above, the moving average method can be utilized to reduce random noise under low-speed measurement conditions. Through the moving average of 10 points, the proposed method could better distinguish the continuous sinusoidal motions. 

## 4. Discussion

According to Equation (1), the measurement resolution can be improved by increasing the distance (*L*) between beams 1 and 2. This can achieved by simply increasing the size of the GCC sensor or decreasing the grating period. However, a decrease in the grating period would induce the ray-cutting problem in the diffracted beams owing to the limited size of the GCC sensor. Therefore, a trade-off exists between the sensor size and grating period. Additionally, the minimum grating period is limited by the critical angle of the corner cube.

In a multi-dimensional system, the effect of the pitch and yaw on the roll angle should be considered. According to Equation (1), the distance *L* between beam 1 and beam 2 is equal to *D*tan(*θ*_+1_)+ *D*tan(*θ*_−1_). Here, the diffracted angle (*θ*_+1_ and *θ*_−1_) depends on the yaw angle, *α*_y_, which should be calculated using other methods and used for compensation. Notably, if the yaw angle is small, the value of *D*tan(*θ*_+1_) + *D*tan(*θ*_−1_) remains nearly unchanged. In this case, the effect of the yaw angle on the roll angle measurement is less than that of random noise, and it can be ignored. In addition, the variation of the pitch angle, *α*_x_, leads to the same displacement of beam 1 and beam 2 in the *Y*-direction (Δy_1_ and Δy_2_). According to Equation (1), the effect of the pitch angle on the roll angle measurement can be eliminated via differential measurements.

In our previous studies, using an optical frequency comb as the light source combined with a GCC sensor, precise and rapid measurements of the absolute distances, yaw, and pitch angles could be achieved by resolving the interferometric phase spectra of the corresponding diffraction beams [23,24,25]. Combined with the method proposed in this paper, the proposed technique could be used to simultaneously measure six degrees of freedom. Future studies will focus on the further expansion of the designed system to enable dynamic six-degree-of-freedom measurements. In this study, considering only the roll angle and lateral displacement measurements, the mode-locked laser in the proposed method can be replaced by other stable laser sources. In addition, a certain diameter of the incident beam was employed in the proposed method to ensure the desired measurement ranges of the axial distance, yaw, and pitch angles. When the GCC sensor is only used as a roll angle sensor, the function of the beam reducer can be realized by reducing the diameter of the fiber collimator. In this case, the resulting experimental setup would be more compact.

## 5. Conclusions

In conclusion, we demonstrated a GCC-based roll angle sensor. A parallel dual beam was naturally constructed by combining a diffraction grating and corner cube. The roll angle could be easily obtained through the differential detection of the two parallel beam positions. We evaluated the accuracy, stability, and resolution of the proposed method. Compared with a commercial autocollimator over the range of 500 arcsec, the residuals were maintained within ±2 arcsec, with an STD of 1.37 arcsec. In addition, stability experiments were also performed, which demonstrated that the STD of the roll angle jitter was lower than 0.46 arcsec over 5 min. The proposed method achieved a measurement resolution of 0.8 arcsec using the GCC sensor. Using such a compact target, sub-arcsec-level roll angle measurements with a response speed of 300 Hz can be achieved. As the two measurement beams from the GCC sensor are always parallel to the incident beam regardless of the target rotation and movement, the proposed technique has potential applications in large-scale metrology over long stand-off distances.
